# All-trans retinoic acid upregulates VEGF expression in glioma cells in vitro

**DOI:** 10.7555/JBR.27.20120048

**Published:** 2012-12-19

**Authors:** Chen Liang, Shiwen Guo, Ling Yang

**Affiliations:** a Department of Neurosurgery, the First Affiliated Hospital of Medical College of Xi'an Jiaotong University, Xi'an, Shaanxi 710061, China;; b Department of Aeromedical Physical Examination, Xi'an Civil Aviation Hospital, Xi'an, Shaanxi 710061, China.

**Keywords:** All-trans retinoic acid (ATRA), vascular endothelial growth factor (VEGF), glioma, hypoxia-inducible factor-1α (HIF-1α), angiogenesis

## Abstract

All-trans retinoid acid (ATRA) is one of the most potent and most thoroughly studied differentiation inducers that induce the differentiation and apoptosis of glioma cells. However, the effect of ATRA on angiogenesis of glioma remains poorly understood. We examined the effect of ATRA on the expression of vascular endothelial growth factor (VEGF) in different glioma cell lines and investigated the underlying mechanism, intending to partially reveal the effects of ATRA on angiogenesis of glioma. Glioma cells were treated by ATRA at 5 and 10 µmol/L. The *VEGF* mRNA transcript levels were determined by real-time RT-PCR and the protein levels of VEGF in glioma cells were evaluated by Western blotting assays. Moreover, hypoxia-inducible factor-1α (*HIF-1α*) mRNA expression was analyzed by using real-time RT-PCR. After treatment with 5 and 10 µmol/L ATRA, the *VEGF* mRNA transcript levels in glioma cells increased remarkably, compared with that in the control group, and the relative protein expression of VEGF was also up-regulated. Meanwhile, the *HIF-1α* mRNA expression also increased. ATRA increases the expression of VEGF in glioma cells at both transcriptional and translational levels.

## INTRODUCTION

Despite the use of the best therapeutic regimens available, the prognosis of malignant glioma, the most common form of primary brain tumors, remains unsatisfactory. All-trans retinoic acid (ATRA) is one of the strongest and most thoroughly studied differentiation inducers. Several studies have shown that ATRA can induce the differentiation and apoptosis of a variety of glioma cells[1], including glioma stem cells (GSCs)[2],[3], a highly tumorigenic and therapy-resistant tumor subpopulation. These results showed that ATRA has the therapeutic potential for patients with glioma. However, as one of the most important influencing factors, angiogenesis plays a crucial role in the formation, development and recurrence of glioma, but the effects of ATRA on angiogenesis of glioma remain unknown. In this study, we examined ATRA effects on angiogenesis by detecting the expression of the vascular endothelial growth factor (*VEGF*) gene in two different glioma cell lines U87 and SHG44. The results provided a better understanding of the mechanisms involved in the therapeutic effects of ATRA for malignant glioma.

## MATERIAIS AND METHODS

### Reagents

ATRA (Sigma-Aldrich Corp., St. Louis, MO, USA) was dissolved in DMSO at 0.01mol/L and stored in light protected vials at –20°C as stock solution. This stock solution was diluted in media to obtain the desired concentrations before use. All experiments were performed under low-light conditions to minimize ATRA photoisomerization. Anti-human VEGF antibody was purchased from Epitomics (Burlingame, CA, USA).

### Cell Culture

Human glioma cell lines U-87MG and SHG44 were purchased from the Cell Resource Center, Chinese Academy of Sciences (Shanghai, China). U87 cells were cultured in Dulbecco's modified Eagle's medium (DMEM, Hyclone, Logan, UT, USA) supplemented with 5% fetal bovine serum (FBS, Hyclone) in 5% CO_2_ at 37°C. SHG44 cells were cultured in RPMI1640 (Hyclone) and supplemented with 5% FBS (Hyclone) in 5% CO_2_ at 37°C.

### Real-time PCR

U87MG and SHG44 cells (3×10^5^ cells/well) seeded in 6-well plates were incubated in media containing different concentrations of ATRA (0, 5 µmol/L and10 µmol/L) for 24 hours. Cells were then lysed and the total RNA was isolated by using RNA fast 200 Kit (Fastage) according to the manufacturer's instructions. RNA was reverse-transcribed by PrimeScript RT Master Mix (TaKaRa, Otsu, Japan). Real-time PCR was performed using SYBR Premix Ex TaqTMII (TaKaRa) and analyzed with Bio-Rad iQ5 software version 2.0. Gene expression was compared using the ∆cycle threshold (∆Ct=Ct_Target_-Ct_β-actin_) method, where *β-actin* was taken as the endogenous reference gene. Change in gene expression was evaluated by the 2^−∆∆Ct^ method[4].All primers were designed and synthesized by TaKaRa Co. and the sequences are listed in ***Table 1***.

### Western blotting assays

Logarithmically growing cells cultured in 25 mL cell culture flasks with ATRA at different concentrations (0, 5 µmol/L and 10 µmol/L) were harvested for assays. The cells were washed twice with phosphate-buffered saline (PBS) and then scraped on ice in 300 µL RIPA lysis buffer with 1 mmol/L PMSF. Lysates were clarified by centrifugation, and samples were boiled in 1×sodium dodecyl sulfate-polyacrylamide gel electrophoresis (SDS-PAGE) sample loading buffer, resolved by SDS-PAGE, and transferred to polyvinylidene fluoride (PVDF) membranes. The membranes were probed with anti-VEGF antibody (Epitomics Bio, Burlingame, CA, USA) diluted in Tris-buffered saline containing 0.02% Tween 20. After wash in Tris-buffered saline containing 0.02% Tween 20, the membranes were incubated with a secondary polyclonal anti-rabbit IgG antibody conjugated to horseradish peroxidase (Bioworld Technology, OH, USA). Membranes were developed with Super Signal West Pico chemiluminescence reagent (Thermo Scientific, USA).

**Table 1:** The sequences of primers used for real-time PCR

Gene	Primer sequence	Product length
*HIF-1α*	Forward 5′-TCTGGGTTGAAACTCAAGCAACTG- 3′	150 bp
	Reverse 5′- CAACCGGTTTAAGGACACATTCTG-3′	
*VEGF*	Forward 5′-TCACAGGTACAGGGATGAGGACAC- 3′	72 bp
	Reverse 5′-CAAAGCACAGCAATGTCCTGAAG-3′	
*β-actin*	Reverse 5′-CAAAGCACAGCAATGTCCTGAAG-3′	186 bp
	Reverse 5′-CTAAGTCATAGTCCGCCTAGAAGCA-3′	

### Statistical analysis

Values were calculated as mean±SD and data were analyzed by the SPSS 17.0 software (SPSS Inc., Chicago, IL, USA). One-way ANOVA was used to compare the groups, and LSD test was conducted for comparison between the groups thereafter. *P* < 0.05 was considered statistically significant.

## RESULTS

### ATRA induces *VEGF* mRNA transcription in glioma cells

**Fig.1:**
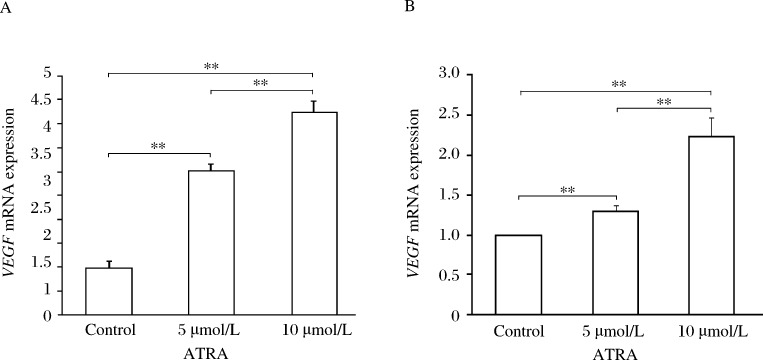
Effects of ATRA on *VEGF* mRNA expression in different glioma cell lines. A: *VEGF* mRNA expression in U87 glioma cells. B: *VEGF* mRNA expression in SHG44 glioma cells. Significant upregulation of *VEGF* mRNA expression was found in the two different glioma cell lines treated with ATRA after 24 hours in different concentrations as compared with that in the control group, **P < 0.01.

We treated the glioma cells with different concentrations of ATRA for 24 hours and then examined the *VEGF* mRNA transcript levels by real-time RT-PCR. We found that compared to the control cells, the *VEGF* mRNA transcript levels were significantly upregulated in the two glioma cell lines treated with ATRA. At 5 µmol/L ATRA, the levels of *VEGF* mRNA transcripts increased 3.02 and 1.30 fold in U87 and SHG44 glioma cells, respectively. At 10 µmol/L ATRA, the *VEGF* mRNA transcripts increased 4.25 fold in U87 glioma cells and 2.24 fold in SHG44 glioma cells ***(Fig.1)***.

### ATRA induces VEGF protein expression in glioma cells

**Fig.2:**
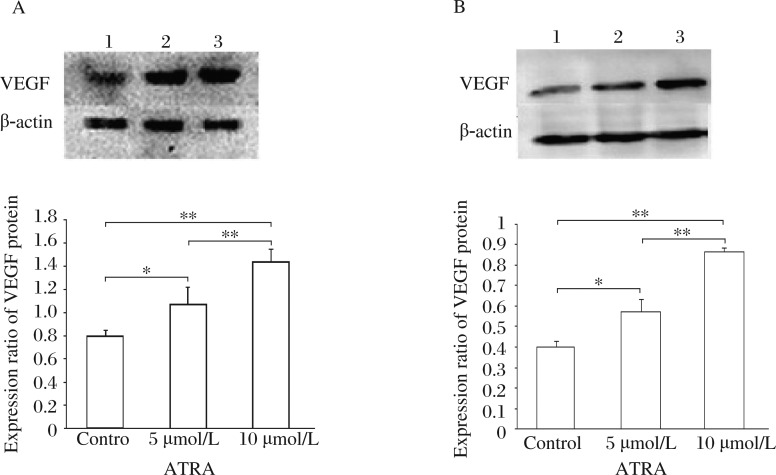
VEGF protein expression in different glioma cell lines. A: VEGF protein expression in U87 glioma cells. B: VEGF protein expression in SHG44 glioma cells. Lane 1: the control group; lane 2: the 5 µmol/L ATRA group; lane 3: the 10 µmol/L ATRA group. VEGF protein expression in glioma cells was also up-regulated by ATRA (*P < 0.05, **P < 0.01).

Consistent with the increase in mRNA transcription levels, the level of VEGF protein expression was also upregulated in glioma cells treated with ATRA. The ratios of VEGF level to that of β-actin were 1.07±0.14 and 0.57±0.06, respectively, in U87 glioma and SHG44 cells treated with 5 µmol/L ATRA. These ratios were even higher in the cells treated with 10 µmol/L ATRA (1.44±0.11 µmol/L in U87 and 0.87±0.01 µmol/L in SHG44) ***(Fig.2)***.

**Fig. 3:**
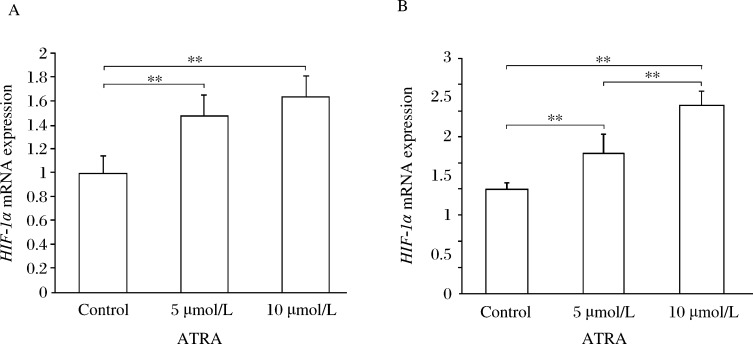
Induction of *HIF-1α* mRNA expressions in U87MG cells by ATRA. A: *HIF-1α* mRNA expression in U87 glioma cells. B: *HIF-1α* mRNA expression in SHG44 glioma cells. ATRA can significantly increase the expression of *HIF-1α* mRNA in glioma cells. The difference between the 5 µmol/L ATRA group and the 10 µmol/L ATRA group in SHG44 glioma cells was statistically significant. But there was no significant difference between the 5 µmol/L ATRA group and the 10 µmol/L ATRA group in U87 glioma cells (**P < 0.01).

### ATRA induces *HIF-1α* *m*RNA transcription in glioma cells

Since VEGF is well known to be regulated by HIF-1α, the effect of ATRA on *HIF-1α* mRNA transcription in glioma cells was examined by real-time PCR. As shown in ***Fig 3***, ATRA significantly increased the level of *HIF-1α* mRNA in glioma cells. There was a significant difference in the *HIF-1α* mRNA transcript levels between the 5 µmol/L ATRA group and the 10 µmol/L ATRA group in SHG44 glioma cells (*P* < 0.05). But there was no significant difference between the 5 µmol/L ATRA Group and the 10 µmol/L ATRA group in U87 glioma cells (*P* > 0.05).

## DISCUSSION

VEGF is a key regulator of angiogenesis and plays an important role in the oncogenesis, progression and recurrence of glioma[5]. Although several studies have reported that the effects of ATRA on the expression of VEGF, the conclusion remains controversial. Some reports have demonstrated that ATRA can inhibit VEGF expression in human gastric cancer cells[6], whereas stimulatory effects of ATRA on the expression of VEGF have been reported in many other studies[7]-[9]. Discrepancies among these studies may be due to the differences in methodology, cell types, experimental conditions and ATRA concentrations used. However, the effects of ATRA on VEGF expression of glioma remain unknown. In this study, we found that ATRA significantly increased VEGF expression in glioma cells at both the transcriptional and translational levels. Moreover, we also found a dose-dependent relationship between the expression level of *VEGF* and ATRA concentration. Our study thus first demonstrated the stimulatory effects of ATRA on *VEGF* mRNA and protein expression in glioma cells.

As HIF-1α is well known as a key regulator of VEGF expression[10], we examined the effects of ATRA on HIF-1α expression in glioma cells. We observed that ATRA significantly induced *HIF-1α* mRNA expression in U87 and SHG44 glioma cell lines. This result was similar to the previous results[11], so we speculate that the upregulation of HIF-1α expression may play a role in the upregulation of VEGF expression in glioma cells. However, the speculation still needs more experimental evidence to support.

The mechanisms of the ATRA-induced HIF-1α expression in glioma cells are still not entirely clear. Recently, researchers have reported that some influencing factors can regulate HIF-1α expression via the MAPK or PI3K signal pathway[12],[13]. Meanwhile, it has also been reported that ATRA can regulate the expression of downstream genes via the MAPK-related rapid phosphorylation pathway[14]. It is therefore speculated that HIF-1α induced by ATRA in U87 cells is partly related to the MAPK pathway. In addition to upregulating the expression of HIF-1α, ATRA may also act through some other pathways to regulate VEGF expression in glioma cells. Some researchers have reported that ATRA can directly induce VEGF expression via the retinoc acid receptor (RAR) signaling pathway[7]-[9], which may be involved in the regulation of VEGF expression of ATRA in treated glioma cells.

As VEGF plays an important role in angiogenesis, the upregulation of VEGF expression in glioma cells induced by ATRA may stimulate angiogenesis of glioma and further promote tumor growth, which requires further investigations. As a therapeutic strategy, ATRA still has some disadvantages in this respect. In conclusion, ATRA can upregulate the expression of VEGF in U87 glioma cells. ATRA may therefore have some disadvantages in the treatment of glioma and the anti-angiogenesis combined therapy may have some more beneficial effects in glioma treatment.
